# An Oxymetazoline-Based Nasal Solution Removes Bacteria–Blood Debris on Dental Surfaces and Has Antimicrobial Activity Toward *Streptococcus mutans*

**DOI:** 10.3390/ijms26031242

**Published:** 2025-01-31

**Authors:** Robert S. Jones, Morgan Annina Pride, Dhiraj Kumar

**Affiliations:** 1 Division of Pediatric Dentistry, Department of Developmental and Surgical Sciences, School of Dentistry, University of Minnesota, Minneapolis, MN 55455, USA; 2 North Carolina Agricultural and Technical State University, Greensboro, NC 27411, USA; mapride@aggies.ncat.edu; 3 Division of Basic Sciences, Department of Diagnostic and Biological Sciences, School of Dentistry, University of Minnesota, Minneapolis, MN 55455, USA

**Keywords:** oxymetazoline, *Streptococcus mutans*, antimicrobial, dental pulp

## Abstract

Background: An over-the-counter vasoconstrictive nasal solution with oxymetazoline (NS-OXY, 0.05%) has the potential to be used as a dental pulpal hemostatic medicament. A molecular engineering approach examined NS-OXY and its molecular constituent’s antimicrobial and blood biomass removal efficacy. Methods: An ex vivo cavity model was developed where standardized prepared teeth were exposed overnight to a model dentinal caries pathogen, *S. mutans*, and then exposed to sheep’s blood for 10 min, which simulated a pulpal exposure. Cavity preparations were rinsed with OXY (0.05%), benzalkonium chloride (BKC-0.025%), NS-OXY (with OXY-0.05% and BKC), ferric sulfate (20%;ViscoStat, FS), and distilled water (DI). For examining the bactericidal effect of NS-OXY, a disk diffusion antimicrobial assay was used where *S. mutans* was grown (20 h) on brain heart infusion (BHI) w/0.5% glucose agar plates and exposed to the treatment groups. Results: NS-OXY-treated samples had a lower residual bacterial or blood biomass than FS (*p* = 0.003). The diffusion test showed that NS-OXY, BKC, and FS had zones of inhibition greater than 10 mm, with NS-OXY having higher activity against *S. mutans* than FS (*p* = 0.0002), but lower than BKC (*p* = 0.0082). Conclusions: NS-OXY may be considered as a dental hemostatic agent after traumatic and carious pulpal exposure owing to NS-OXY’s antimicrobial and vasoconstrictive properties.

## 1. Introduction

Sympathomimetic agents, such as oxymetazoline (OXY) and xylometazoline (XYL), selectively agonize alpha-adrenergic receptors, which initiates hemostasis and reduces tissue edema [1]. Nasal solutions with OXY (NS-OXY) and XTL (NS-XYL) have been used over-the-counter (OTC) as decongestants for decades in the United States and throughout the world [2]. NS-OXY, available in the USA, and NS-XYL, available in many parts of the world, have additional off-label use in the management of bleeding during surgical otorhinolaryngology procedures and intubated anesthesia [3]. Recently, OXY was incorporated in an intranasal dental anesthetic based on its highly effective vasoconstriction, which prolongs anesthesia duration [4]. OXY has also been investigated recently for potential use in gingival bleeding and retraction [5]. Even with large adoption across medicine and recent uses in dentistry, NS-OXY has not been studied in the context of dental pulpal management.

Traumatic or caries-related pulp exposure can occur in both the primary and adult permanent teeth. NS-OXY has the potential to be used off-label to manage pulpal bleeding during traumatic and caries-related pulp exposures and allow for material placement in direct pulp-capping or pulpotomies [6]. As a sympathomimetic agent, OXY mimics epinephrine and other catecholamines. OXY-induced vasoconstriction surpasses the activity of epinephrine through activation of alpha(α)-1 postsynaptic receptors associated with arteriole smooth muscles [7]. In addition to the nasal mucosa, dental pulp tissue also contains α-1 receptors that are activated by the sympathetic nervous system [8]. As a α-1 receptor agonist, OXY can create hemostasis in damaged pulpal tissue via vasoconstriction of smooth muscles associate with arterioles and venules [8,9]. This is similar to OXY-mediated decongestion where nasal tissue edema is reduced via vasoconstriction [10]. As a selective α-1 receptors agonist, OXY has low activity on beta-adrenergic receptors that increase heart rate [11]. This selectivity adds to the safety profile of NS-OXY in children and adults for use as a nasal decongestant and hemostatic agent in surgery [12]. The widespread use of NS-OXY and OTC designation suggests that the solution may be biocompatible with dental pulp tissue [1,2].

The principal aim in pulpal management is to achieve effective hemostasis while avoiding a large blood clot that may potentially lead to slower pulpal healing [13]. Larger blood clots can harbor bacteria and surgical debris and reduce intimate tissue contact with a biocompatible capping material placed over the pulp [14]. In addition to providing effective hemostasis that may limit the buildup of a large blood clot, NS-OXY products contain compounds that may eradicate oral bacteria that can contaminate the blood clot. NS-OXY contains benzalkonium chloride (BKC) and edetate disodium (EDTA), which are antibacterial compounds [6].

In situations where clinicians are faced with managing pulp tissue during the surgical removal of caries or after traumatic injury, oral bacteria ingress from saliva or the caries lesion complicates final restoration management [15]. Immediate hemostasis is required prior to the application of a biocompatible pulp-capping material. Short term hemostasis may improve the material setting of the pulp-capping material that protects the pulp from the oral cavity [16]. Additionally, longer-term success of pulpal management requires creating an ideal restorative seal and leaving little to no residual bacteria, such as *Streptococcus mutans* that can compromise the restoration and directly enter the pulpal blood stream. While not historically viewed as an endodontic pathogen [17], a recent PCR primer based identification study elucidated that *S. mutans* may have an underappreciated role in pulpal necrosis, with an abundance of nearly 70% in root canal spaces of necrotic teeth [18]. Recent investigation has also implicated *S. mutans* in pulpal cell infection and systemic infections, such as endocarditis [19,20,21].

In the present study, the effects of NS-OXY on removing aggregate *S. mutans* and blood biomass debris from dentin and enamel were assessed. This study used a novel ex vivo assay to compare NS-OXY biomass cleansing efficacy and its constituent ingredients (OXY and BKC) against ferric sulfate, which is often used for vital pulpotomy management. Since NS-OXY is a commercial formulation with potential off-label use, a molecular engineering approach reconstructed the effects of individual ingredients of the whole product. To study the direct bactericidal effect of NS-OXY and potential risk reduction in residual pulpal infection and caries, a disk inhibition assay was used to examine the treatment groups against *S. mutans* growth.

## 2. Results

**Figure 1:**
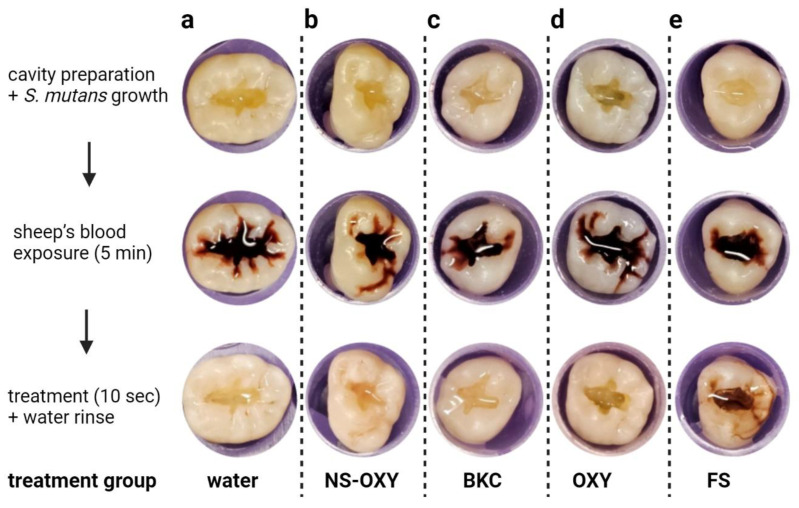
Ex vivo bacteria and pulpal exposure model using a cavity preparation in dentin followed by overnight incubation in presence of *S. mutans* in BHI media and subsequent exposure to sheep’s blood (5 min). Bacteria–blood was exposed to treatment (10 s) and rinsed with 2 mL of distilled water. Residual debris was stained and quantified with crystal violet and spectroscopic analysis. Treatment groups were (**a**) control-distilled water, (**b**) NS-OXY that contained 0.05% OXY, (**c**) 0.025% BKC, (**d**) 0.05% OXY, and (**e**) 20% ferric sulfate (FS). FS was the only group that left visible residual staining.

**Figure 2:**
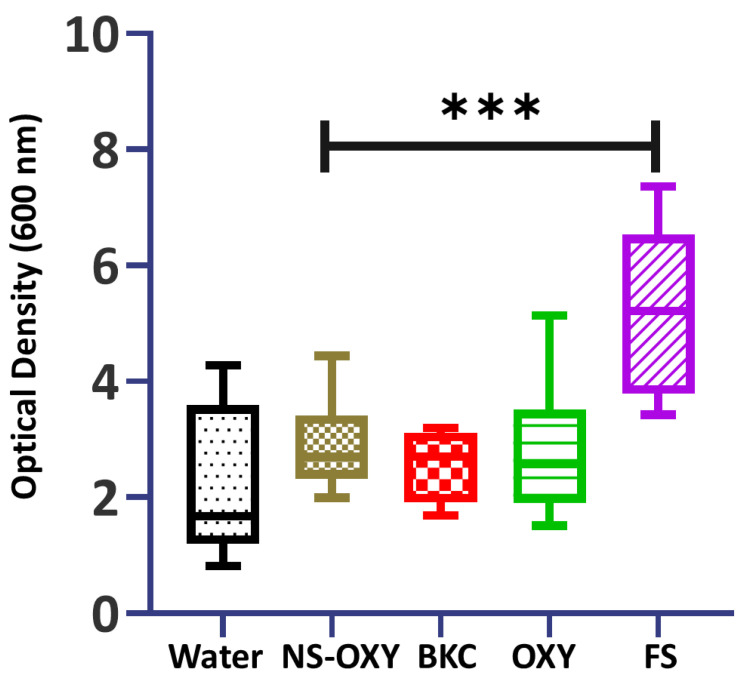
Residual biomass of bacteria and sheep’s blood was quantified with a crystal violet assay that measured the absorbance units (optical density) with a spectrophotometer at 600 nm. *** *p* = 0.003.

Steps of the ex vivo model are shown in Figure 1 where *S. mutans* was grown in the presence of teeth with standardized tooth cavity preparations and then exposed to sheep’s blood (SB) for 5 min. The OTC nasal spray with 0.05% oxymetazoline (NS-OXY), BKC (0.025%), OXY (0.05%), and the control (sterile distilled water) with a subsequent 2 mL water rinse removed the bacteria–SB debris efficiently from the cavity preparations, leaving little residual biomass. On visual inspection, and shown in Figure 1, 20% FS left a brownish residual biomass even after the water rinse step. Crystal violet staining and biomass quantification revealed that FS had a substantially higher residual biomass after water rinsing than NS-OXY, BKC, OXY, and distilled water (Figure 2).

**Figure 3:**
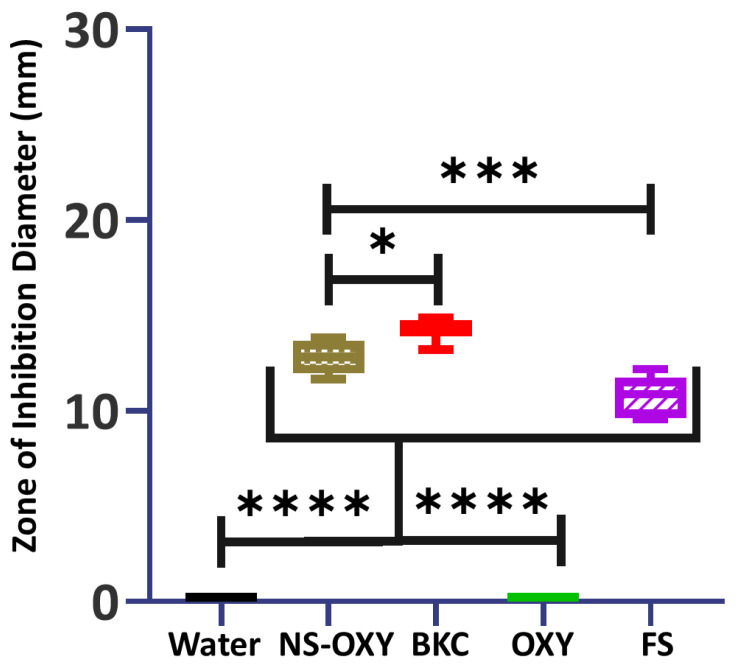
Quantifying the zone of inhibition (ZOI) diameters. Box plots of the diameters and standard deviations of the ZOIs for each test sample against *S. mutans.* (n = 6). Both water and OXY had zero ZOI for all samples. * *p* = 0.0082; *** *p* = 0.0002; **** *p* < 0.0001.

**Figure 4:**
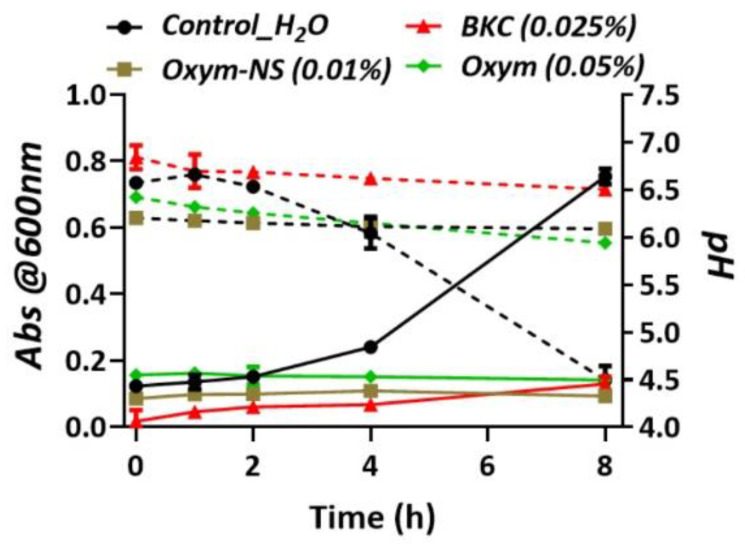
Growth inhibition assay with *Streptococcus mutans’* (n = 3) growth over 8 h in BHI media measured by optical density (solid line) and pH (dotted line).

In the disk diffusion susceptibility test, NS-OXY, BKC (0.025%), and 20% FS inhibited *S. mutans*’ growth after 20 h on BHI agar (Figure 3). BKC treatment had a larger zone of inhibition than NS-OXY and FS. NS-OXY had statistically higher antimicrobial activity toward *S. mutans*. BKC, NS-OXY, and FS demonstrated greater than 10 mm zone of inhibition. OXY and deionized water demonstrated no inhibition of the growth of *S. mutans* on BHI-agar plates. In contrast, OXY inhibited *S. mutans* growth during 8 h in BHI broth (Figure 4). This inhibitory effect was comparable to BKC (0.025%) and 0.01% NS-OXY. NS-OXY had to be diluted to 0.01% since it could not be pre-concentrated as a commercial formulation.

## 3. Discussion

This laboratory-based study demonstrated that an OTC vasoconstrictive nasal solution with oxymetazoline (NS-OXY, 0.05%) had greater biomass removal and antimicrobial activity than ferric sulfate (FS). NS-OXY demonstrated higher antimicrobial activity towards *S. mutans* than FS. BKC, at a concentration of 0.025%, showed a larger zone of inhibition of *S. mutans* growth than NS-OXY. OXY, by itself, produced no zone of inhibition against agar cultures of *S. mutans*, which was analogous to the water control in the disk susceptibility assay. Broth cultures of *S. mutans* are more susceptible to antimicrobial effects, and OXY, along with BKC, and NS-OXY, all inhibited planktonic growth of *S. mutans*.

The results of the present study support that BKC, which is an ingredient in NS-OXY, is likely the main compound exerting a substantial antimicrobial effect on *S. mutans*. This allows NS-OXY to exert antimicrobial activity with closer physiological pH (5.81) than ferric sulfate (2.55). BKC, a cationic organic quaternary ammonium compound, exerts a membrane disruption action against *S. mutans* [22]. BKC has been explored previously in dentistry as an additive in adhesives and cements due to the compounds broad antimicrobial activity and anti-matrix metalloproteinase activity [23,24,25,26,27,28]. In NS-OXY, BKC is labeled as an inactive ingredient, since it does not contribute to the labeled use of NS-OXY, which is decongestion via vasoconstriction of the nasal vasculature. BKC and EDTA, which are also found in NS-OXY, are preservatives that increase the shelf life of the NS-OXY bottle and reduce bacterial contamination when NS-OXY is used intranasally and is in contact with nasal bacteria. This is especially important when used as a decongestant spray in repeated dosing inside the anterior portion of the nasal cavity.

For a potential dental application, NS-OXY can be potentially applied to exposed pulp tissue with a carrier, such as a cotton ball or micro-brush. NS-OXY is commonly available to clinicians during dental work under general anesthesia since the medicament is used to manage nasal obstruction and bleeding during nasal intubation by anesthesia teams [12]. The antimicrobial activity of NS-OXY has the potential to sterilize the pulp exposure while providing hemostasis through activation of alpha-adrenergic receptors (α-1) within pulp tissue. While the microbiota of deep caries is diverse [29], *S. mutans* is highly cariogenic in caries and is a model organism for testing antimicrobial activity among Gram-positive bacteria. While *S. mutans* may not be historically associated with recurrent apical periodontitis, most of these studies have focused on endodontically treated teeth with intact coronal restorations [17]. Several newer studies demonstrate that *S. mutans* may be associated with direct infection of pulpal cells and systemic infections, including endocarditis, and may penetrate into the blood stream via the pulpal tissue [18,19,20,21].

While this study demonstrated that FS had antimicrobial activity toward *S. mutans,* FS was limited in removing bacteria and blood debris in the ex vivo tooth model. In this model, a water rinse step occurred after all the treatment applications including FS. FS left bacteria–blood debris residue that was not removed by a subsequent water rinse. FS agglutinates, or clumps together, blood proteins and iron-protein complexes, which are key to the mechanism of repairing damaged blood vessels [30]. This chemical reaction is more pronounced at an acidic pH (2.55). Future research is needed to understand whether the low pH and inadequate debris–blood removal of FS, with residual iron-protein complexes, may lead to resorption, which is often cited as a side effect in FS pulpotomies [31,32,33]. NS-OXY left little bacteria–blood residue biomass when used in the bacteria and pulpal model. Additional studies are needed to assess the biocompatibility of NS-OXY with a more biocompatible pH of 5.81.

As demonstrated in this study, NS-OXY does not leave enamel and dentin tissue with any residual color that would interfere with esthetics. NS-OXY can also potentially be used to sterilize the overlaying dentin and enamel cavity preparation. This cavity disinfection can be further explored since this activity provides an additional benefit over FS, which also leaves a brownish color residue while providing antimicrobial activity. FS has limited usage in the management of anterior pulp exposures (e.g., Cvek partial pulpotomy) or cavity disinfection.

The results of this study do not perfectly correlate the NS-OXY antimicrobial activity with the 0.025% BKC results. BKC showed approximately 30% higher antimicrobial activity than NS-OXY. There are some potential reasons for this discrepancy. First, the concentration of BKC in NS-OXY is proprietary. This work estimated its potential concentration to be 0.025% [6]. In addition, BKC is not a single compound, like chlorhexidine. BKC is a blend of alkylbenzyldimethylammonium chlorides (ABAC), and the exact formulation blend is also proprietary. In the present work, a blend of 70% benzyldimethyldodecylammonium chloride and ~30% benzyldimethyltetradecylammonium chloride was used to optimize solubility and was reproducible in combination with a commercially available product from a major distributor. Further studies could explore the various OXY and ABAC formulations that maximize solubility, tissue biocompatibility, and antimicrobial activity. Further research into optimizing BKC with NS-OXY would be a full-scale venture since ABAC can be chosen with different chain lengths that can affect solubility, membrane disruption, cavity cleaning, and tissue compatibility. The current OTC NS-OXY may have less antimicrobial activity than 0.025% BKC, but it still has the potential to disinfect the cavity preparation and pulpal exposure and effectively remove debris and blood. Future studies can investigate NS-OXY effect on other bacterial species including multispecies cultures that may be more resistant to antibiotics.

In terms of potential systemic effects, NS-OXY has been used safely as an OTC nasal spray in both children and adults for several decades [1,2]. OXY is a selective α-1 receptor agonist with limited beta-receptor effects on heart tissue. Reported adverse hemodynamic effects attributed to NS-OXY relate to extensive and undefined soaking of nasal pledgets during nasal surgical applications or copious applications in very young children or use in individuals with complex medical issues [34,35,36]. Defined dosages of NS-OXY in surgical nasal applications have shown minimal systemic effects, and it has been speculated that self-limiting vasoconstriction reduces systemic effects via slow absorption [3,37]. There is no current evidence that a single application of OXY causes excessive vasoconstriction that could lead to distal end necrosis in distal tissues [6]. Only an animal tail study with periodic application (3x/daily) for 4 weeks has shown distal tissue necrosis from extensive vasoconstriction caused by OXY [38]. As a pulpal medicament, it is estimated that 0.05 mL of 0.05% NS-OXY delivered via cotton ball or micro-brush may be required for hemostasis during a pulpotomy [6]. Serious adverse events in children age 5 and younger have occurred at volumes substantially greater (1–2 mL) [39]. Further investigation is needed regarding the safety profile of NS-OXY as a pulpal medicament in children and adults. More work is also needed to explore the pulpal compatibility and antimicrobial activity of other imidazoline derivatives, such as XYL. NS-OXY possesses properties that suggest it can be used off-label as a hemostatic agent in managing carious and traumatic dental pulp exposures.

## 4. Materials and Methods

**Reagents and materials.** An OTC nasal solution with 0.05% oxymetazoline (NS-OXY) was purchased (Afrin^®^ original, Bayer AG, Berlin, Germany), and the measured pH was 5.81. A custom 0.05% oxymetazoline (OXY) solution was prepared from stock OXY (Cayman Chemical, Ann Arbor, MI, USA) and deionized (DI) water with a measured pH of 4.57. A 0.025% benzalkonium chloride (BKC, Millipore Sigma, Burlington, MA USA), also known as alkylbenzyldimethylammonium chloride, was prepared with DI water with a final pH of 4.70. According to the manufacturer’s information, the BKC was a mixture of different chain length alkylbenzyldimethylammonium chlorides: ~70% benzyldimethyldodecylammonium chloride CH_3_(CH_2_)_11_N(Cl)(CH_3_)_2_CH_2_C_6_H_5_ and ~30% benzyldimethyltetradecylammonium chloride CH_3_(CH_2_)_13_N(Cl)(CH_3_)_2_CH_2_C_6_H_5_. The solutions were stored at 4 °C until used at room temperature in the assays described below. Syringes of 20% ferric sulfate (FS, ViscoStat™) were purchased from Ultradent (South Jordan, UT, USA) with a measured pH of 2.55. Specialized porous disks (Cytiva Whatman^TM^, Marlborough, MA, USA, 05-711, 6 mm) were used in the diffusion assay. Due to infection control restrictions, human blood was substituted with sheep blood (SB, R54012, Fisher Scientific, Waltham, MA, USA), which is a blood type that is available and used in specialized bacterial media. For bacterial growth experiments, brain heart infusion broth (Difco-237200, BD, Franklin Lakes, NJ, USA) was used with commercial bottled water.

**Ex vivo bacteria and pulpal exposure model.** Extracted teeth were obtained as pathological specimens from routine dental care without patient identifiers. The collection process was exempt from full review by the University of Minnesota Institutional Review Board, based on the USA Code of Federal Regulations 46.104(4ii). Using extracted non-carious teeth, a simulated ex vivo pulpal access cavity model was created, in which a tooth cavity preparation was made and exposed to *S. mutans* and later sheep’s blood. Subsequent exposure of the treatment groups (NS-OXY, OXY, BKC, FS, and sterile distilled water (control)) measured bacterial–blood debris removal and residual biomass.

The ex vivo model used standardized tooth cavity preparation using a carbide bur. The final cavity depth and width was 2.5 mm × 3.5 mm with the depth of the preparation exposing the underlying sound dentin with sidewall enamel. The model standardized a simulated pulpal access cavity. The rationale was that the high variability of the 3D geometry of real pulpal space made standardizing the exposed area more difficult across treatment groups.

*S. mutans* grew overnight in BHI broth supplemented with 0.5% glucose. A bacterial inoculum of *S. mutans* was added to fresh 10-mL suspensions of BHI-glucose broth in 20-mL scintillation vials. Teeth with the standardized simulated pulpal access cavity were placed inside the vials, which were then placed in the anaerobic chamber described above and shaken (100 rpm). Broth cultures were decanted after overnight incubation, and samples were exposed to sheep blood (SB) for 5 min by placing 30–50 µL volume in the tooth cavity. The standardized bacteria–SB exposure simulated the pulp exposure scenario.

Simulating in vivo pulpal medicament application, teeth exposed to bacteria–SB were washed with respective treatments (n = 9, sterile bottled water, NS-OXY, BKC, OXY, and FS) using 2.0-mL volume (500 µL aliquots, 10 s hold) followed by 2.0-mL distilled water rinse. Quantifying the residual biomass, 500 µL of 0.1% crystal violet (CV) solution in water was added to 24-well plates. Tooth samples were transferred, upside down, into wells and exposed to CV for 15 min at 37 °C. Teeth were removed and subjected to a water rinse until no CV stain (blue color) was detected in the water rinse. The teeth were exposed to 500 µL 1M acetic acid solution that solubilized the CV-stained biomass complex in a 24-well plate at room temperature. Next, a 200 µL CV–acetic acid suspension was pipetted into a 96-well plate and the stain intensity was measured using a microplate spectrophotometer (Epoch, Biotek, Winooski, VT, USA) at 600 nm.

**Bacterial growth inhibition assays.** *Streptococcus mutans* (ATCC 700610) cultures were grown in BHI media with 0.5% glucose anaerobically at 37 °C without shaking in an anaerobic chamber (Coy Laboratory Products, Grass Lake, MI, USA) with an atmosphere of 5% CO_2_ and 95% N_2_. To test treatment inhibition, a disk inhibition assay was performed. Plates of BHI agar media with 0.5% glucose were prepared. A bacterial inoculum of *S. mutans* (300 µL) (OD, 0.5 at 600 nm) was spread on plates using a disposable L-shaped spreader. BHI agar with *S. mutans* plates were dried at room temperature for up to 15 min. Diffusion assay disks (n = 6) had 20 µL of treatment reagents added. Treatment groups (n = 6) included NS-OXY, OXY (0.05%), BKC (0.025%), and FS (20%). Disks were dried for 10 min and gently pressed into the BHI agar with *S. mutans* plates. The plates were incubated anaerobically, as described above, for 20 h. Disks were visually assessed and measured for the absence or degree of the zone of inhibition around treatment disks [40]. Zone of inhibition distances (mm) from the disks were measured using digital images, analyzed with ImageJ (version 1.54h, National Institutes of Health, Bethesda, MD, USA).

BHI broth cultures of *Streptococcus mutans* were exposed to three of the above treatment groups (n = 3) in addition to a non-treatment control. Ferric sulfate interferes with the spectrophotometric measurement of optical density (OD) growth when monitoring liquid cultures and was not tested in this second assay. OXY (0.05%) and BKC (0.025%) were prepared with the BHI media by pre-concentrating the reagents prior to a 1:5 dilution (25 mL). Since NS-OXY was a commercial formulation, a 1:100 dilution was made with BHI (0.01% NS-OXY). An initial inoculum of *S. mutans* (OD ~0.10–0.15 at 600 nm) was added to 25 mL of BHI in 50 mL centrifuge tubes and grown in an anaerobic chamber. Aliquots were sampled periodically during 24 h to examine growth (OD) and media pH.

**Statistical Analysis.** To test for differences between treatment groups (NS-OXY, OXY, BKC, FS, and sterile distilled water (control)) on *S. mutans* growth and residual biomass, a one-way ANOVA was performed using Prism (GraphPad, Boston, MA, USA). Holm–Sidak’s multiple comparison testing was used to assess specific differences between treatment groups. Box and whisker graphs depicted the 25th and 75th percentile (box outline), median (horizontal line in the box), and 95% confidence intervals for the median (error bars-whiskers).

## 5. Conclusions

Based on the results of this in vitro study, an OTC nasal spray with 0.05% oxymetazoline (NS-OXY) showed higher antimicrobial activity toward *S. mutans* and superior bacteria–blood removal than 20% FS. Future studies may examine the potential of NS-OXY to be used as a pulpal medicament that initiates hemostasis and provides antimicrobial activity.

## Data Availability

Data supporting the reported results has been archived by The University of Minnesota Digital Conservancy and found at the data repository for the University of Minnesota (DRUM) (https://hdl.handle.net/11299/269713).

## Abbreviations

The following abbreviations are used in this manuscript:

α-1	alpha-1
ABAC	alkylbenzyldimethylammonium chlorides
BHI	brain heart infusion
BKC	benzalkonium chloride
CV	crystal violet
DI	Distilled
EDTA	edetate disodium
FS	ferric sulfate
NS-OXY	nasal solution containing OXY
OD	optical density
OTC	over-the-counter
OXY	oxymetazoline (XYL)
PCR	polymerase chain reaction
SB	sheep’s blood
XYL	Xylometazoline
ZOI	zone of inhibition
